# Assessment of the results and hematological side effects of 3D conformal and IMRT/ARC therapies delivered during craniospinal irradiation of childhood tumors with a follow-up period of five years

**DOI:** 10.1186/s12885-020-07168-7

**Published:** 2020-07-29

**Authors:** Zoltán Lőcsei, Róbert Farkas, Kornélia Borbásné Farkas, Klára Sebestyén, Zsolt Sebestyén, Zoltán Musch, Ágnes Vojcek, Noémi Benedek, László Mangel, Gábor Ottóffy

**Affiliations:** 1 Clinical Center, Department of Oncotherapy, University of Pécs, Édesanyák útja 17, Pécs, 7624 Hungary; 2 Oncoradiology Center, Uzsoki Hospital, Uzsoki u. 29-41, Budapest, 1145 Hungary; 3 Unicersity of Pécs, Medical School, Institute of Bioanalysis, Szigeti út 12, Pécs, 7624 Hungary; 4 Oncology Unit, Clinical Center, Department of Pediatrics Pécs, University of Pécs, József Attila út 7, Pécs, 7623 Hungary

**Keywords:** Craniospinal irradiation, Medulloblastoma, RapidArc, Childhood cancer

## Abstract

**Background:**

Craniospinal irradiation (CSI) of childhood tumors with the RapidArc technique is a new method of treatment. Our objective was to compare the acute hematological toxicity pattern during 3D conformal radiotherapy with the application of the novel technique.

**Methods:**

Data from patients treated between 2007 and 2014 were collected, and seven patients were identified in both treatment groups. After establishing a general linear model, acute blood toxicity results were obtained using SPSS software. Furthermore, the exposure dose of the organs at risk was compared. Patients were followed for a minimum of 5 years, and progression-free survival and overall survival data were assessed.

**Results:**

After assessment of the laboratory parameters in the two groups, it may be concluded that no significant differences were detected in terms of the mean dose exposures of the normal tissues or the acute hematological side effects during the IMRT/ARC and 3D conformal treatments. Laboratory parameters decreased significantly compared to the baseline values during the treatment weeks. Nevertheless, no significant differences were detected between the two groups. No remarkable differences were confirmed between the two groups regarding the five-year progression-free survival or overall survival, and no signs of serious organ toxicity due to irradiation were observed during the follow-up period in either of the groups.

**Conclusion:**

The RapidArc technique can be used safely even in the treatment of childhood tumors, as the extent of the exposure dose in normal tissues and the amount of acute hematological side effects are not higher with this technique.

## Background

Statistically, tumors of the central nervous system rank second in terms of incidence among childhood neoplastic diseases in most European countries, including Hungary [1]. Radiotherapy is extremely important as part of postoperative treatment. Full craniospinal axis irradiation (CSI) is performed postoperatively in medulloblastomas/PNETs and for the treatment of some rarer tumors, for example, atypical rhabdoid tumors or ependymomas that have already been disseminated in the CSF space. During routine craniospinal radiotherapy, the full neural axis is irradiated, most commonly at a dose of 35–36 Gy, followed by a boost treatment to the tumor nest at a minimum dose of 54 Gy. These doses are described by the Hungarian National Cranial Protocol for Childhood Tumors [2–7].

**Fig. 1:**
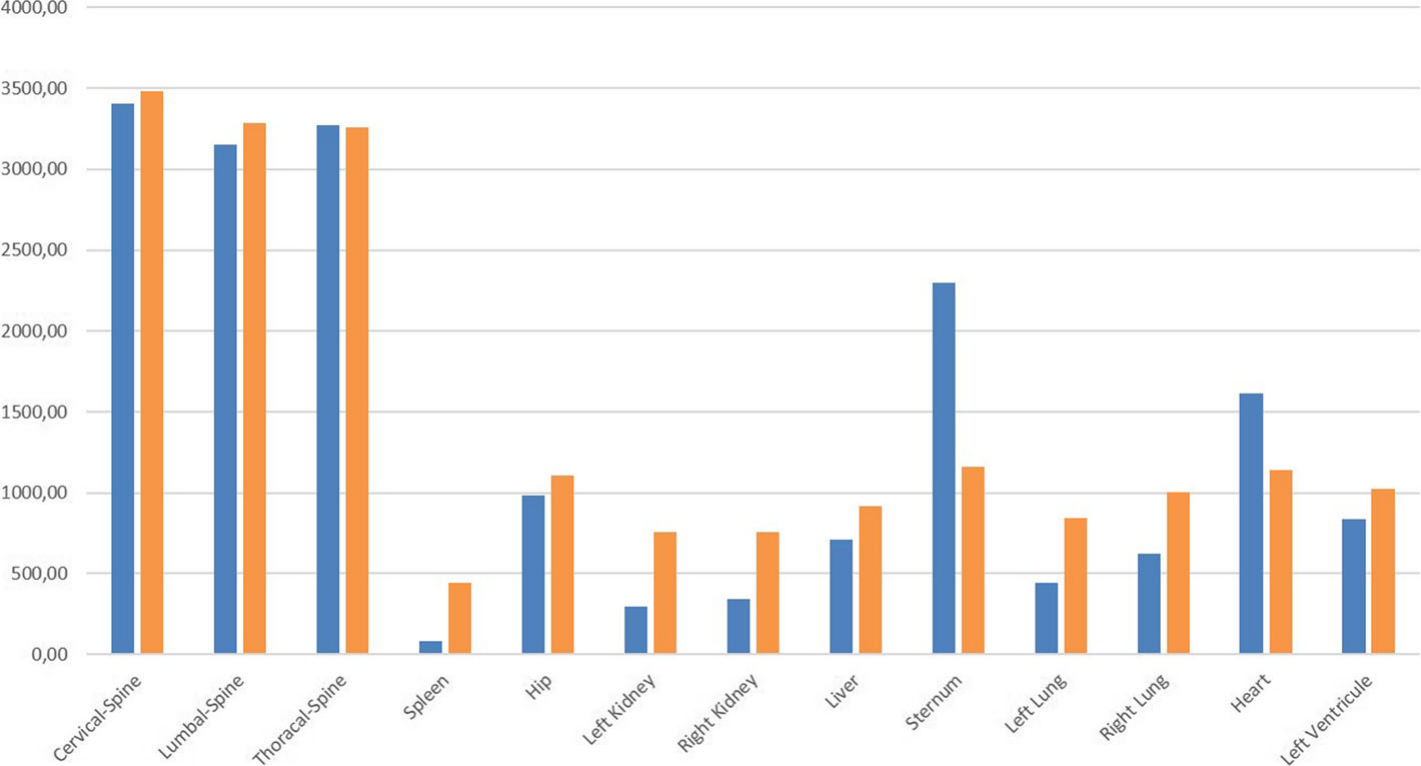
OAR dose exposures (cGy) during the treatments carried out with the two radiotherapeutic modalities. 3DCRT in blue and IMRT/ARC therapy in orange

**Fig. 2:**
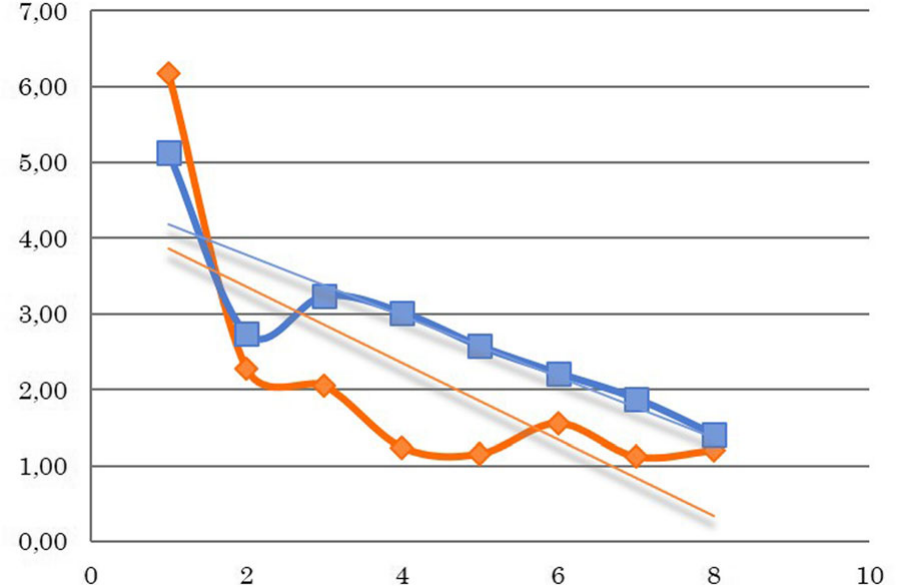
Neutrophil counts for all patients (G/l) during the treatment weeks. The decrease in the weekly mean value of neutrophil granulocytes during the treatment. A significant decrease can be observed during the treatment weeks; however, there is no difference between the two groups. (Orange: 3D-conformal plan, Blue: IMRT/ARC plan)

**Fig. 3:**
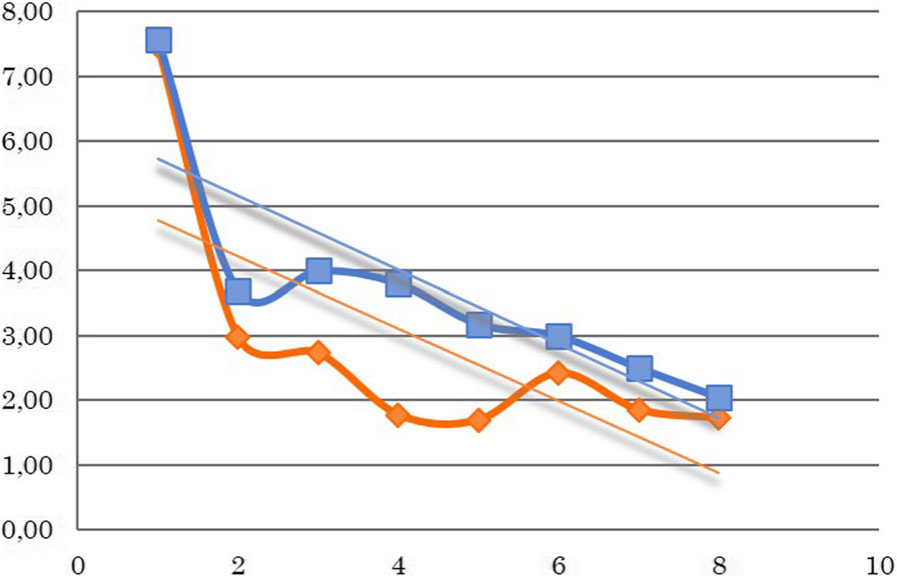
White blood cell counts for all patients (G/l) during the treatment weeks. The decrease in the weekly mean value of white blood cell counts during treatment. A significant decrease can be observed during treatment weeks; however, there is no difference between the two groups. (Orange: 3D-conformal plan, Blue: IMRT/ARC plan)

**Fig. 4:**
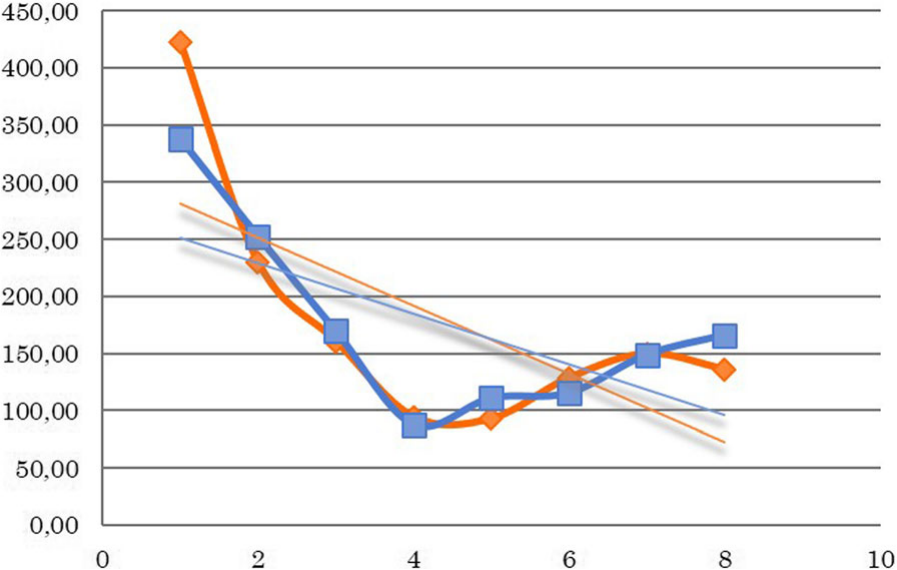
Platelet counts for all patients (G/l) during the treatment weeks. The decrease in the weekly mean value of platelets during treatment. A significant decrease can be observed during treatment weeks; however, there is no difference between the two groups. (Orange: 3D-conformal plan, Blue: IMRT/ARC plan)

**Fig. 5:**
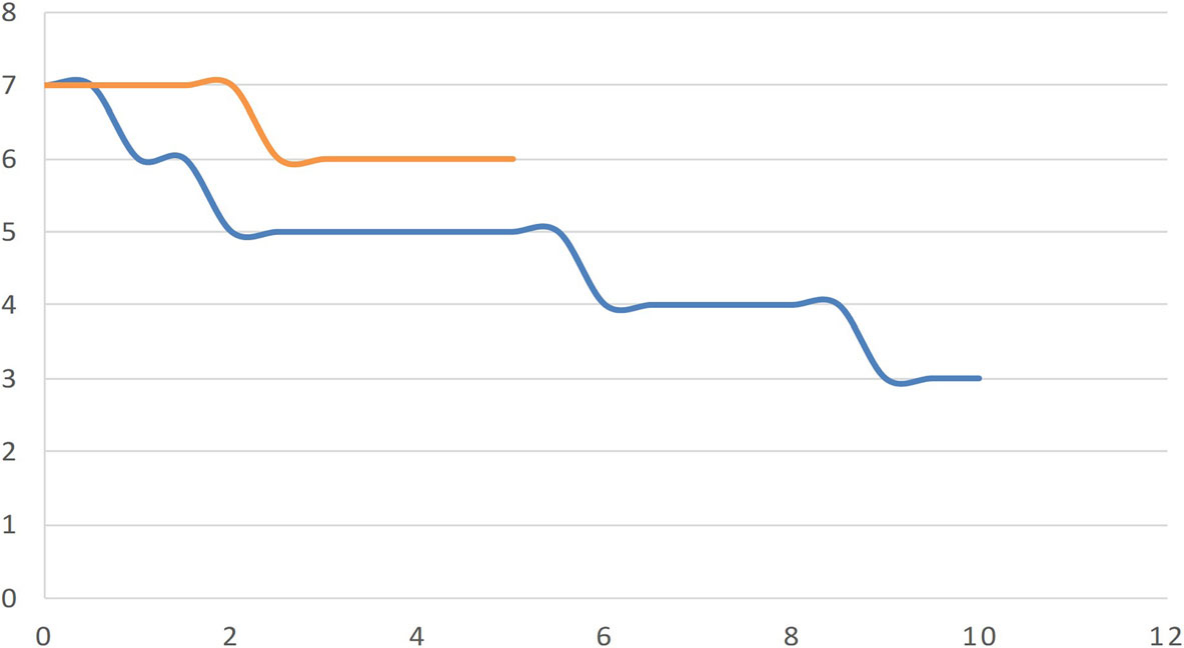
Progression-free survival. All patient curves over the years. 3DCRT in blue and IMRT/ARC in orange

**Fig. 6:**
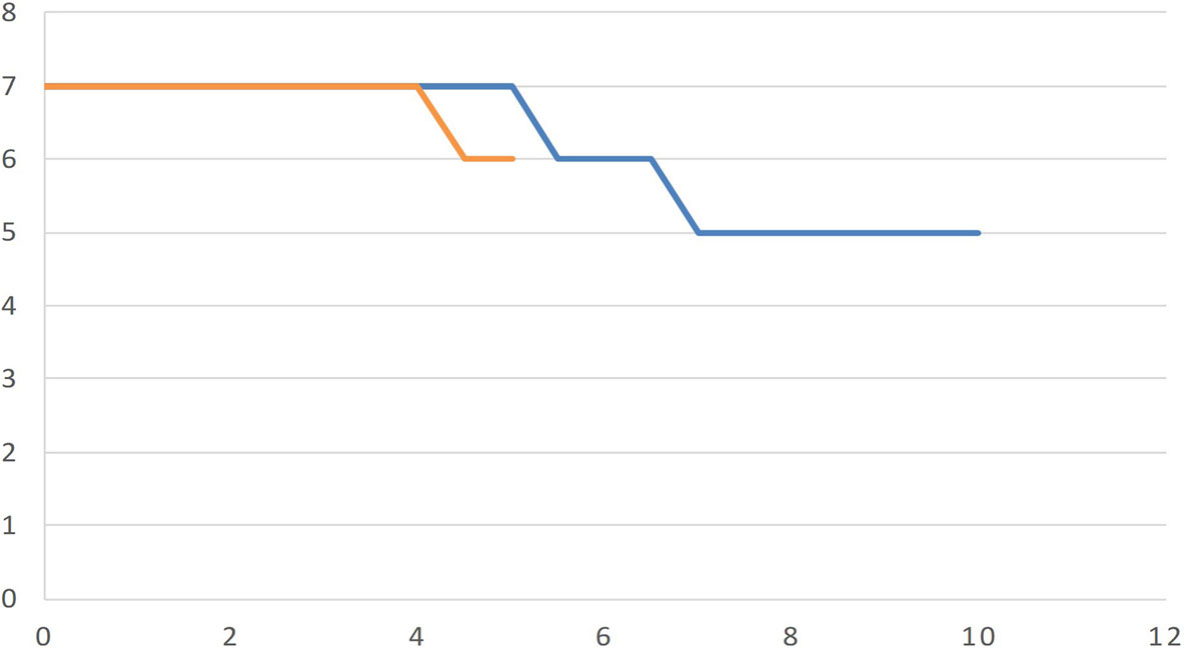
Overall survival data. All patient curves over the years. 3DCRT in blue and IMRT/ARC in orange

Acute side effects may occur during radiotherapy and may lead to the discontinuation of treatment. These side effects may be of neurological or hematological origin; however, other types of side effects may also occur. Side effects affecting quality of life can be expected following doses delivered to organs not located in the central nervous system.

The question of side effects arises in conjunction with advances in modern radiotherapeutic technology, such as intensity modulated radiation therapy, but mainly in the area of therapeutic radiation treatment, i.e., whether the integrated dose exposure, which theoretically can be even higher, caused by the field entries from multiple directions or the more extensive radiation exposure, although with a lower dose, of normal tissues and organs causes more acute - predominantly hematological - toxicities. Naturally, it is also a question of whether the dose exposure of the parenchymal organs is genuinely higher when using these new techniques.

Thus, we assessed the effects of both types of treatment techniques in terms of both the bones important for hematopoiesis and the parenchymal organs. In addition, based on the changes in hematological parameters obtained during the treatment, we attempted to draw some conclusions concerning additional bone marrow toxicity.

Positioning is essential during CSI treatment due to the extent of the treated volume; therefore, another objective is to decrease the daily uncertainty of the setup. IMRT/ARC therapy and image guidance offer simpler and more precise treatment delivery, obligatory on such occasions. Another purpose of these novel technologies might have been to decrease the acute side effects related to treatment, since even the airways (trachea, bronchi) can receive a lower dose rate when using IMRT/ARC. The experience gathered with IMRT/ARC is presented in this paper.

## Methods

Full CSI was carried out in 14 children and young adults with primary intracranial brain tumors, with a mean age of 14.64 years (3–33 years of age) at our institute between 2007 and 2014. We included each and every consecutive pediatric brain tumor patient who was treated during the study period. Each patient signed an informed consent form to participate in the retrospective data analysis. Guardians or parents signed for patients under the age of 18. In accordance with Hungarian regulations, no ethical approval was obtained for the analysis of our data. The treatment of patients before 2011 was performed with the 3D conformal technique and field alignment in a prone position. Subsequently, patients were treated with IGRT and the RapidArc technique in a prone position. 3D conformal treatments were delivered with the Elekta Eclipse PreciseTS device, while the RapidArc treatments were carried out with the Varian Novalis TX linear accelerator. Retrospectively, seven patients were identified separately in both groups, and our patients were followed in a partially prospective manner. Based on the histological types, predominantly medulloblastoma (11 cases), PNET (1 case), atypical rhabdoid tumor (1 case) and glioblastoma (1 case) were observed. All patients, except the glioblastoma patient, underwent primary surgery and adjuvant chemotherapy in accordance with the Hungarian National Cranial Protocol. A vacuum bed and head mask were used during positioning. It was decided to use an open-face mask during the treatment in a supine position; additionally, in order to be able to reproduce the positioning of the entire body, the patient’s arms were fixed beside their body. During radiotherapy, a median of 35.2 Gy (30.4–36.8 Gy) was delivered to the whole spine and the skull, followed by a posterior fossa boost of a median dose of 19.8 Gy (19.2–24 Gy). The CTV for the spine was defined cranially from the C1 vertebral body caudally to the S2 vertebral body. The vertebral body and spinous process in an antero-posterior direction and the transverse foreman latero-laterally were used as borders. A CTV PTV expansion of 4 mm was used. For posterior fossa irradiation, the primary tumor was defined as the GTV and extended by 1 cm to the CTV. The tumor bed was included in this CTV. A PTV was generated with a 3 mm margin from the previous structure.

Regarding the retrospective assessment of acute toxicity, the results of the follow-up laboratory tests performed during treatment were reviewed. The counts of white blood cells, platelets and red blood cells as well as the levels of hemoglobin and hematocrit were analyzed during treatment. Version 25 of SPSS software was used for the calculations. Repeated ANOVA tests were performed for all values except for the difference between the age values and during the calculation of hemoglobin levels, where independent sample t-tests were used. Furthermore, assessments were completed regarding the exposure dose of the organs-at-risk to determine whether IMRT/ARC therapy would eventually be associated with a higher exposure dose, predominantly regarding the hematopoietic organs. The entire bony spine was divided into three segments; thus, the cervical, thoracic and lumbar spine segments were contoured. In addition, the sternum, pelvic bones, spleen and liver were contoured. The doses delivered to the heart, left ventricle, kidneys and lungs were also determined to assess exposure doses affecting the quality of later life. It was also noted that, on many occasions, it was necessary to suspend treatment for over 1 week due to the acute side effects caused by the treatment. Our study also reviewed the treatment results using data obtained from the local pediatric oncological care center after the treatment in order to evaluate the progression-free and overall survival data. We also used long-term care data to check whether any delayed organ toxicity associated with radiotherapy had occurred in any child.

## Results

The mean age of the patients in the 3D conformal population was 15.71 years (± 9.69 years) compared with 13.57 years (± 11.77 years) in the IMRT/ARC arm. The independent sample t-test showed no significant difference between the mean age (*p* = 0.710).

The first point of analysis of the side effects caused by radiotherapy was the extent of exposure dose in the normal tissues. The mean exposure dose of the organs at risk responsible for the hematopoietic side effects in the case of the 3D conformal and IMRT/ARC treatments were as follows: cervical spine: 3408/3484 cGy, thoracic spine: 3271/3261 cGy, lumbar spine: 3152/3288 cGy, sternum: 2299/1156 cGy, pelvic bone: 987/1104 cGy, spleen: 81/460 cGy, and liver: 708/917 cGy. No significant differences were observed in the bones near the target area between the two types of radiation therapy; however, the exposure dose of the sternum decreased and that of the spleen increased during IMRT/ARC.

The exposure doses of the nonhematopoietic organs at risk were as follows: heart: 1612/1140 cGy, left ventricle: 827/1025 cGy, right kidney: 343/757 cGy, left kidney: 298/755 cGy, right lung: 623/1003 cGy, and left lung: 441/845 cGy. An increase regarding the organs at risk was detected with Arc therapy; however, these changes are well within the tolerability criteria according to the QUANTEC dose charts (Fig. 1).

While the exposure dose of organs at risk is caused by a single direct field directed at the spine when using the 3D conformal technique, the characteristics of the rotating field of Arc irradiation during IMRT/ARC therapy means that more organs at risk may be affected by a lower dose. Thus, a slight dose increase may be experienced with this technique compared to the 3D conformal technique; however, this is tolerable.

After analyzing weekly changes in the laboratory parameters, the following conclusions were made despite the low number of cases. The repeated measures ANOVA test revealed the following regarding the observed laboratory parameters. The total white blood cell counts significantly decreased compared to the baseline values over the weeks (*p* = 0.0029), while the neutrophil counts initially increased then also decreased (*p* = 0.007). The same significant decrease was observed in the platelet counts (*p* = 0.0004). No changes were observed in the red blood cell counts (*p* = 0.107) or in the hematocrit levels (*p* = 0.140); however, a slight difference was observed in the hemoglobin levels (*p* = 0.045). Nevertheless, no significant differences were observed between the two groups regarding the total white blood cell count (*p* = 0.449), neutrophil (*p* = 0.754), platelet (*p* = 0.815), red blood cell (*p* = 0.506), hematocrit (*p* = 0.489) or hemoglobin (*p* = 0.360) parameters (Figs. 2, 3 and 4).

Two cases of grade 3 leukopenia were seen in the 3D conformal arm, while only grade 1 side effects were noted in the IMRT/ARC arm. However, several cases of grade 2 thrombocytopenia were observed in the IMRT/ARC arm, and the results of these patients did not essentially affect the mean values of the corpuscular cell parameters for the given week. One week breaks in the therapy became necessary on two occasions in each of the two groups, either due to leukopenia or thrombocytopenia. Furthermore, no delayed organ toxicities were noted.

We have been following our patients for 12 years. The median follow-up duration in the 3D conformal group was 10 years compared to 5 years in the RapidArc group.

In terms of progression-free survival, the development of local recurrence or new organ manifestations in patients with a poorer prognosis affected the development of the curves in both groups (Fig. 5).

There was no significant difference between the development of the overall survival curves of the two populations in the first five years (Fig. 6).

## Discussion

CSI irradiation is a challenging treatment, not only due to patient age but also because of the many challenges of its practical application. While planning 3D conformal radiotherapy, it is difficult to align the entire cranial irradiation with the field treating the spine and to align the spinal fields with each other. The cranial field is usually covered by two lateral fields, while the spinal fields consist of single posterior fields. The development of “hot spots”, dose inhomogeneities, increases at the alignment points, thus increasing the risk of overdosing [8–11]. Sebestyén et al. demonstrated the technique used on eight patients at their institute to avoid overdosing. By using segments in the field, no overdosed areas developed at the points of field alignment [12]. This may be reduced by using the intensity modulate technique (IMRT) [13]. Using the IMRT, Kuster et al. managed to decrease the homogeneous dose distribution while increasing coverage of the target area and protection of the organs at risk [14].

With further advancements in radiotherapeutic techniques and planning options and with volumetric arc therapy (VMAT) becoming increasingly widespread, it became necessary to study how much gentler this treatment modality is compared to conventional stationary field IMRT. Rolina et al. analyzed the plans of ten patients. They improved the coverage of the target area by using the VMAT technique; however, this did not result in significant differences. No remarkable differences were seen in terms of the exposure doses of the organs at risk between the two techniques [15]. These results were supported by other studies conducted at other institutes [16–18]. In the SIOP-E-BTG group study, the same cases were sent to 15 institutes for planning to compile the best 3D-CRT, IMRT, VMAT and proton therapeutic plans. The modern radiotherapeutic techniques resulted in improvements in dose conformity and dose homogeneity compared to 3D-CRT. The dose exposure of organs at risk also improved; however, significant differences were only obtained with proton therapy [19].

Hideghéty et al. assessed the benefits and disadvantages of prone and supine patient positioning in 12 patients. No differences were observed regarding dose homogeneity or coverage. However, the supine position was more advantageous in terms of patient comfort and achieving a simple treatment [20].

The side effects of the treatment may be acute or delayed. In the current study, we essentially dealt with the acute side effects and sought an explanation for their development. While using IMRT and other modern techniques in the St. Claire study, the dose limits of organs at risk were not approached compared to 3D-CRT; thus, they believed that the side effects may decrease [21]. During the prospective study of Cox conducted between 2010 and 2014, acute side effects were analyzed in ten patients. Gastrointestinal side effects, such as vomiting and diarrhea, occurred predominantly during the treatments. However, these side effects are well tolerated with appropriate supportive care, unlike the significantly more therapy-resistant side effects of alopecia and headache [22]. As an effect of dose modulation during IMRT, the dose delivered towards the abdominal organs is well controllable; therefore, the side effects are also more tolerable [14]. In the HIT-91 study, according to the description of Kortman et al., treatment interruptions became necessary due to the occurrence of myelosuppressive side effects. Notable (> grade 3) myelosuppression was seen in 35% of patients who received chemotherapeutic regimens before and after their radiotherapy and in 19.3% of patients who only received maintenance therapy. The hematological side effects were especially prolonged in young adults. By eliminating the direct field, the dose of the sternum - an organ at risk - was successfully reduced by 57% using IMRT [23]. This was also supported by our results, as the dose for the sternum was 2299/1156 cGy. We demonstrated the safety of rotating field arc radiation therapy, with no remarkable myelosuppressive side effects observed.

The acute side effect of bone marrow suppression is typical during treatment. The work of Sung Zong-Wen outlined that a large area of tissue is affected by a relatively low dose during VMAT. In addition, the main side effect in treated patients was hematological toxicity, which did not exceed the decrease beyond the grade (Gr) 3 value [24]. Wong et al. observed hematological toxicity of the following magnitude in 14 patients during VMAT. Leukopenia Gr 2: 11%, Gr 3: 26%, Gr 4: 63%, Anemia Gr 2: 89%, Thrombocytopenia Gr 1–2: 16%, Gr 3: 26%, and Gr 4: 37% [25]. Kumar et al. conducted a study involving four institutes between 2011 and 2014 that analyzed the hematological causes of therapy discontinuation in 52 patients. Treatment was discontinued if a grade 2 side effect developed and was continued if grade 1 side effects appeared. Irradiation of the spine had to be interrupted in 73.1% of patients due to leukopenia in 92% of cases and thrombocytopenia in 2.6% of cases, while both were responsible in 5.3% of cases [26]. In our study, we encountered milder side effects both in the 3D conformal arm and the IMRT/ARC arm.

Salloum et al. processed mortality and morbidity data from patients treated for medulloblastomas between 1970 and 1999; thus, these data covered three decades. The median time from diagnosis in the 1311 enrolled patients was 21 years. The 15-year mortality rates were 23.2 and 12.8% in patients treated in the 70 s and 90 s, respectively; the mortality rates due to recurrence were 17.7 and 9.6%, respectively [27]. Altogether, the role of advancing and developing techniques was highlighted; we also set a similar objective for our study. Similarly, good results were achieved using these advanced techniques during the follow-up of our patients. Although the overall survival curves in our study developed in a very similar way, only a trend can be suggested. This result is a consequence of the low number of patients. Our study has some limitations due to the very small sample size and heterogeneity of the cohort.

## Conclusions

The analysis of our patients’ treatments highlighted that there was no notable difference between the two treatment modalities in terms of the normal tissue dose exposure; indeed, the dose exposures to certain organs and tissues can even be reduced markedly with the use of modern technology. IMRT/ARC therapy can be carried out more reliably and easily from the perspective of both patients and radiotherapy technicians. Although there were a small number of cases, there was no difference in the decrease in laboratory parameters between the two groups. Therefore, from the point of view of hematologic side effects, IMRT/ARC treatment is also safe. In our experience, the different dose exposures do not markedly affect the laboratory parameters, nor do they cause acute complications. Longer follow-up intervals and a larger number of patients are necessary to assess delayed side effects.

## Data Availability

The datasets used and/or analyzed during the current study are available from the corresponding author upon reasonable request.

## Abbreviations

3D: Three-dimensional
IMRT/ARC: Intensity modulated radiotherapy with moving gantry
CSI: Craniospinal irradiation
SPSS: Statistical Package for the Social Sciences
PNET: Primitive neuro-ectodermal tumor
Gy: Gray
GTV: Gross tumor volume
CTV: Clinical target volume
PTV: Planning target volume
ANOVA: Analysis of variance
cGy: centi Gray
QUANTEC: Quantitative Analyses of Normal Tissue Effects in the Clinic
OAR: Organs at risk
3D-C: 3D-conformal plan
RA: IMRT/ARC plan
VMAT: Volumetric arc therapy
3D-CRT: Three-dimensional conformal radiotherapy
HIT-91: Hirmtumor-91 study
Gr: Grade
SIOP-E-BTG: International Society for Pediatric Tumor – Europe – Brain Tumor Group
